# Atomic Force Microscopy Study of the Temperature and Storage Duration Dependencies of Horseradish Peroxidase Oligomeric State

**DOI:** 10.3390/biomedicines10102645

**Published:** 2022-10-20

**Authors:** Irina A. Ivanova, Maria O. Ershova, Ivan D. Shumov, Anastasia A. Valueva, Yuri D. Ivanov, Tatyana O. Pleshakova

**Affiliations:** Institute of Biomedical Chemistry, 119121 Moscow, Russia

**Keywords:** horseradish peroxidase, atomic force microscopy, enzyme aggregation, enzymatic activity, protein storage

## Abstract

This paper presents an investigation of the temperature dependence of the oligomeric state of the horseradish peroxidase (HRP) enzyme on the temperature of its solution, and on the solution storage time, at the single-molecule level. Atomic force microscopy has been employed to determine how the temperature and the storage time of the HRP solution influence its aggregation upon direct adsorption of the enzyme from the solution onto bare mica substrates. In parallel, spectrophotometric measurements have been performed in order to estimate whether the HRP enzymatic activity changes over time upon the storage of the enzyme solution. The temperature dependence of the HRP oligomeric state has been studied within a broad (15–40 °C) temperature range. It has been demonstrated that the storage of the HRP solution for 14 days does not have any considerable effect on the oligomeric state of the enzyme, neither does it affect its activity. At longer storage times, AFM has allowed us to reveal a tendency of HRP to oligomerization during the storage of its buffered solution, while the enzymatic activity remains virtually unchanged even after a 1-month-long storage. By AFM, it has been revealed that after the incubation of a mica substrate in the HRP solution at various temperatures, the content of the mica-adsorbed oligomers increases insignificantly owing to a high-temperature stability of the enzyme.

## 1. Introduction

Studying functional and structural properties of proteins represents an actual topic of modern biochemistry. New information about these properties allows one to understand mechanisms of intermolecular interactions and functional roles of proteins in the body, and, consequently, to reveal causes of pathologies in humans.

Enzymes are known to play key roles in the processes occurring in cells, and in the body in general. Functional properties of enzymes are influenced by a number of factors, including temperature, pH, and the presence of inhibitors [1,2]. At that, for some enzymes, there is a correlation between their structural and functional properties [3,4,5].

Temperature is a universal basic factor influencing the functional activity of all types of enzymes. This is why any model, describing the regulation of functional activity of an enzyme, should include a kinetic description of the temperature dependence of the enzyme’s functioning [6]. Generally, thermal denaturation of enzymes occurs due to a destabilization in ionic and hydrophobic interactions, and at the expense of breakage of hydrogen and van der Waals bonds. This denaturation causes conformational changes in the tertiary structure of an enzyme, making it functionally inactive [2,7,8]. The conformational stability of the enzyme structure is influenced by temperature, pH, and various denaturation conditions [9], and is extensively studied, as enzymatic reactions are performed under various conditions, including high temperatures [10]. Temperature represents one of the external factors, which determines the relative content and thermodynamic characteristics of active conformations of an enzyme in equilibrium [11].

The effect of temperature on enzymatic activity is usually studied in order to determine the mechanism of the enzyme functioning [2]. It is known that a change in oligomeric state of an enzyme can lead to the formation of aggregate structures; the latter can have an unfavorable influence on its functional properties [12,13,14]. For instance, in [7], enzyme aggregation was reported to be the primary cause of glucose oxidase (GOD) inactivation. This inactivation can be prevented by changing the microenvironment of the enzyme [8].

In modern biochemistry, novel technologies for the investigation of protein properties are being actively developed. Thus, cryo-(electron microscopy) (cryo-EM) was used for studying the oligomeric state of proteins [15,16]. Methods of computer modeling, including those employing artificial intelligence, should be particularly emphasized. For instance, the capabilities of AlphaFold2 (an open-access product developed in 2021 by Google) focus on the prediction of 3D structures of a protein based on its amino acid sequence, which represents a fundamental problem of modern science [17]. The novel technology of artificial intelligence opens new opportunities for studying biological systems, allowing for the transition from the gene sequence to the protein conformation.

In our present study, we propose the application of atomic force microscopy (AFM) for studying one of the structural properties of a biological macromolecule—namely, its oligomeric state (aggregation state). AFM allows one to obtain data on structural features of a protein, including its oligomeric state [3], under near-native conditions with sub-nanometer height resolution [3,18]. AFM is known to be a powerful tool for studying single-protein molecules and their complexes [18,19,20,21]. AFM was previously employed for the visualization of protein components of cytochrome P450-containing systems and their complexes [19,22,23,24,25], and for the determination of the oligomeric state of HRP under various conditions [12,13,14,26,27,28,29].

In this study, horseradish peroxidase (HRP) has been employed as a model object, as it was characterized in the literature in much detail, allowing us to better explain the experimental results obtained. HRP represents a heme-containing glycoprotein enzyme of plant origin [30,31]. HRP catalyzes the oxidation of a wide range of substrates by hydrogen peroxide. This 44-kDa glycoprotein contains two Ca^2+^-binding sites, one of which is proximal, while another one is distal relative to the heme group. The structure of the HRP globule also includes four S-S bonds, *N*-glycosylation, and numerous hydrogen bonds [30,32,33]. HRP has found numerous applications in medical diagnostic systems [30], biotechnology [34], and analytical chemistry [35,36,37]. This is the reason why the stability of HRP is an important parameter, which determines the applicability of this enzyme [38].

Altikatoglu et al. [9] demonstrated that the temperature dependence of the biocatalytic activity of native HRP in aqueous medium has the form of a bell-shaped curve with the maximum at *T =* 30 °C. At that, the presence of a 75-kDa dextran additive at concentrations of 10, 20, and 30% has a stabilizing effect on the HRP activity at *T* > 25 °C. At room temperature, HRP retains its activity for up to 15 days [39]. Various additives usually exhibit different effects on the surface of the enzyme globule and the charge state of HRP [9]. These effects can change the pH dependencies of the stability and the enzymatic activity of HRP [9]. The use of additives at various pH likely changes the surface charge of the HRP globule and, consequently, leads to a shift in the isoelectric constant of the environment [40].

Herein, AFM is employed for the determination of the aggregation state of HRP adsorbed on the atomically smooth surface of bare mica substrates. The use of the HRP solution under conditions typical for a laboratory biochemical experiment is modeled, and the temperature of the solution and its storage duration are varied. Our study is aimed at the determination of the influence of these solution incubation conditions on the aggregation state of HRP at the level of single molecules. Spectrophotometry has been used as a reference method of HRP enzymatic activity estimation [12,13,14,26,27,28,29]. The latter, however, does not reveal the effects observed by AFM.

We propose a methodological approach, which can find its application in studies of properties of other water-soluble proteins.

## 2. Materials and Methods

### 2.1. Chemicals and Protein

Lyophilized powder of HRP from horseradish was purchased from Sigma (Cat. #6782). A 2,2′-azino-bis(3-ethylbenzothiazoline-6-sulfonate) (ABTS) substrate was purchased from Sigma. Disodium hydrogen orthophosphate (Na_2_HPO_4_), citric acid, and hydrogen peroxide (H_2_O_2_) were all of analytical or higher-purity grade, and were purchased from Reakhim (Moscow, Russia). Dulbecco’s modified phosphate-buffered saline (PBSD) was prepared by dissolving a salt mixture, commercially available from Pierce, in ultrapure water. All solutions used in our experiments were prepared using deionized ultrapure water (with 18.2 MΩ × cm resistivity), obtained with a Simplicity UV system (Millipore, Molsheim, France). A 10^−7^ M (0.1 µM) solution of HRP was prepared by dissolving the above-mentioned commercially available HRP preparation in 2 mM PBSD buffer.

### 2.2. Experiment Design

The influence of storage of the 10^−7^ M HRP solution at a temperature of *T* = 4 °C for a 31-day period on its adsorption behavior on mica substrates was monitored. The AFM and spectrophotometry measurements were performed on the 1st, 7th, 14th, and 28th day of solution storage. By AFM, the effect of incubation temperature (15, 20, 22, 25, 30, 35, and 40 °C) of the 10^−7^ M HRP solution on the HRP adsorption behavior on mica was also investigated. In order to minimize its influence on the conformation of the enzyme macromolecules, and to stabilize their structure, we used a low-salt (2 mM) buffer. We proposed the comparison of the existing data on the enzymatic activity of HRP with the data on the oligomeric state of this enzyme obtained by AFM after their adsorption onto the surface of mica substrates. The HRP sample solutions in the buffer were incubated at various temperatures within the 15–40 °C range. The HRP was directly adsorbed from the sample solutions onto mica substrates, and then it was visualized by AFM.

### 2.3. Sample Preparation for AFM Experiments

For AFM experiments, the following series of samples were prepared.

(1) The series for the determination of the effect of temperature

The initial 0.1 µM HRP solution in 2 mM PBSD buffer was aliquoted into standard 1.7 mL Eppendorf-type test tubes (SSI Bio, Lodi, CA, USA), and stored at 4 °C for 24 h. Then, 7.5 × 15 mm pieces of freshly cleaved bare mica were placed into the tubes and incubated for 10 min at 600 rpm in a Thermomixer Comfort laboratory shaker (Eppendorf, Hamburg, Germany) at either of the following temperatures: 15, 20, 22, 25, 30, 35, or 40 °C. In this way, HRP was allowed to directly adsorb onto mica during the incubation. After the incubation, each mica substrate with adsorbed HRP was rinsed with fresh ultrapure water, and dried in air. For each temperature point studied, the experiments were performed in at least three technical replicates.

(2) The series for the determination of the effect of storage duration

The initial 0.1 µM HRP solution in 2 mM PBSD buffer was aliquoted into standard 1.7 mL Eppendorf-type test tubes (SSI Bio, USA), and stored at 4 °C for either 1, 7, 14, or 28 days. After the storage of the samples, 7.5 × 15 mm pieces of freshly cleaved bare mica were then placed into the tubes and incubated for 10 min at 600 rpm and room temperature in a Thermomixer Comfort laboratory shaker (Eppendorf, Germany). After the incubation, each mica substrate with adsorbed HRP was rinsed with fresh ultrapure water and dried in air. For each storage time studied, the experiments were performed in at least three technical replicates.

(3) Blank experiments

In order to estimate the amounts of nonspecific objects adsorbed onto the substrates, the mica sheets were immersed into 1 mL of either protein-free 2 mM PBSD buffer or ultrapure deionized water, and incubated therein for 10 min at 600 rpm and room temperature in a Thermomixer Comfort laboratory shaker (Eppendorf, Germany). After the incubation, the substrate incubated in the buffer was rinsed with ultrapure deionized water. Both substrates were finally dried in air.

### 2.4. AFM Scanning

The surface of mica substrates with adsorbed HRP was scanned by AFM in intermittent contact mode in air at 25 °C with either a NTEGRA Aura or a Titanium atomic force microscope (NT-MDT, Zelenograd, Russia; the Titanium atomic force microscope pertains to the equipment of “Human Proteome’’ Core Facility of the Institute of Biomedical Chemistry, supported by Ministry of Education and Science of Russian Federation, Agreement No. 14.621.21.0017, unique project ID: RFMEFI62117X0017). NSG10 cantilevers (“TipsNano”, Zelenograd, Russia; tip curvature radius 6–10 nm; resonance frequency 47–150 kHz, force constant 0.35–6.1 N/m) were installed into the microscopes. The scan size was 2 µm × 2 µm (with 256 × 256 resolution), and no less than 16 scans in different substrate areas were obtained for each substrate studied. A TGZ1 grating (Zelenograd, Russia; step height 21.4  ±  1.5 nm) was used for the calibration of the microscopes.

### 2.5. AFM Data Processing

The heights of mica-adsorbed HRP were determined as the heights corresponding to the maxima of the respective distributions of the mica-adsorbed objects with heights *ρ(h)* as reported in [41]:

*ρ(h)* = (*N_h_*/*N*) × 100% (1)

where *N_h_* is the number of AFM-visualized mica-adsorbed objects of the height *h*, while *N* is the total number of mica-adsorbed objects visualized by AFM.

The experimentally obtained dependence (1) was then approximated using the Gaussian function:

(2) $$\rho (h)=\sum^{\;}\rho_{i}\left(h\right)=\overset{2}{\underset{i=1}{\sum }}\frac{Ae^{\frac{-4\;ln(2){\left(h-h_{c}\right)}^{2}}{w^{2}}}}{w\sqrt{\frac{\pi }{4\;ln(2)}}}$$

where *A, h_c_,* and *w* are the parameters varied upon approximation. The maxima of the *ρ(h)* distributions were calculated as the maxima of the approximation Function (2) for each distribution. The analysis of the approximation of the experimentally obtained *ρ(h)* distributions is based on a χ^2^ criterion.

The initial processing of the AFM images obtained (second-order plane subtraction) and the data export to ASCII format were performed with an Image Analysis software (NT-MDT, Zelenograd, Russia) supplied with the atomic force microscopes. The number of objects, visualized in the AFM images obtained, was calculated with a specialized Recognite software (developed in Institute of Biomedical Chemistry in collaboration of Moscow Engineering Physical Institute MEPhI).

### 2.6. Spectrophotometric Measurements

Enzymatic activity of HRP against ABTS was estimated by molecular absorption spectroscopy according to the technique reported by Sanders et al. [42], following the recommendations provided by the manufacturer of the enzyme preparation [43]. Briefly, time dependencies of absorbance *A_405_(t)* of the 10^−9^ M HRP solution in phosphate-citrate buffer (51 mM Na_2_HPO_4_, 24 mM citric acid, pH 5) were recorded at 405 nm for 5 min with an 8453 spectrophotometer (Agilent Deutschland GmbH, Waldbronn, Germany) in the following way. A 30 µL aliquot of 10^−7^ M HRP solution was added to 2.95 µL of buffer in a quartz cell with a pathlength of 1 cm (Agilent Deutschland GmbH, Waldbronn, Germany) and stirred thoroughly. Then, an 8 µL volume of 3% (*w*/*w*) hydrogen peroxide solution was added into the cell, and spectrum acquisition started immediately.

## 3. Results

### 3.1. Blank Experiments

**Figure 1:**
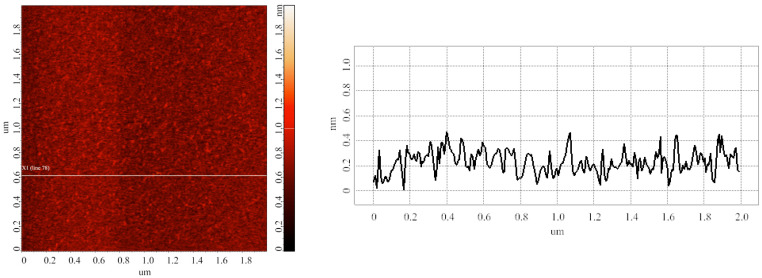
Typical AFM image (**left**) obtained in the blank experiment with the use of pure 2 mM protein-free PBSD buffer. (**Right**) panel displays a cross-sectional profile corresponding to the white line in the AFM image shown on the left. For the AFM image, scan size is 2 µm × 2 µm, and Z scale is 2 nm.

Blank experiments were carried out in order to estimate the heights of objects, which adsorbed onto mica substrates nonspecifically. The substrates were prepared as described in the Materials and Methods (series 3). Figure 1 displays the typical AFM image obtained in the blank experiments.

The image shown in Figure 1 indicates that the heights of objects, nonspecifically adsorbed onto mica, do not exceed 0.6 ± 0.2 nm.

### 3.2. Effect of Incubation Temperature of HRP Solution on Enzyme Aggregation State on Mica

**Figure 2:**
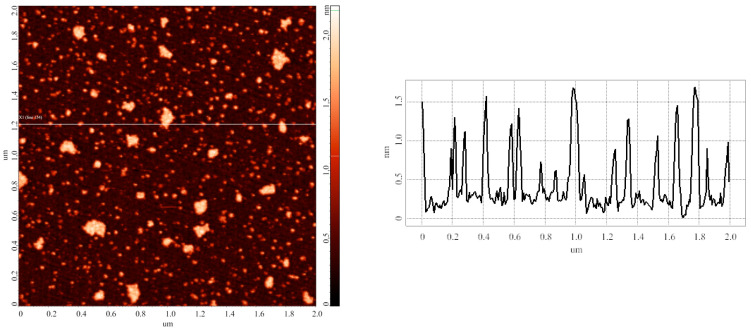
Typical AFM image (**left**) of objects adsorbed on mica substrate after its incubation in 0.1 µM HRP solution in 2 mM PBSD buffer at room temperature (*T* = 25 °C). (**Right**) panel displays cross-sectional profile corresponding to the line in the AFM image. For the AFM image, scan size is 2 µm × 2 µm, and Z scale is 2 nm.

In the first step of the study, the surface of the samples incubated at room temperature (*T* = 25 °C) was visualized. The substrates were prepared as described in the Materials and Methods (series 1). The incubation temperature in this experiment corresponded to that used in routine techniques of laboratory biochemical analysis. This experiment was performed in order to estimate characteristic heights of objects, which can be attributed to HRP molecules. Figure 2 displays the typical AFM image obtained in this step.

The image shown in Figure 2 indicates that both compact and laterally extended objects with heights up to 2 nm are visualized on the mica substrate after its incubation in 0.1 µM HRP solution in 2 mM PBSD buffer at room temperature.

**Figure 3:**
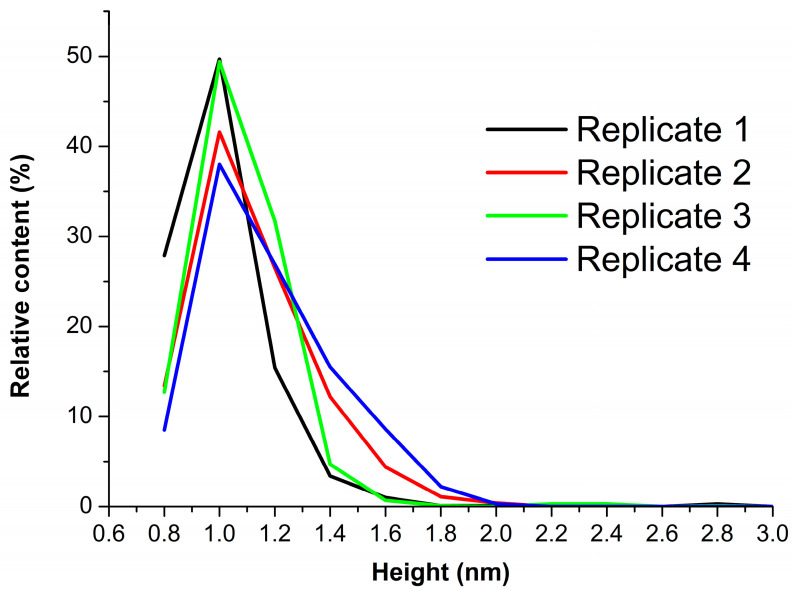
Results of processing the AFM data obtained in four technical replicates of the experiment. Plots of the relative distributions of mica-adsorbed objects, visualized by AFM after the incubation of the substrates in 0.1 µM HRP solution at room temperature (*T* = 25 °C), with height. Curves obtained in different technical replicates, performed under the same conditions, are shown in different colors.

Figure 3 displays the results of processing the data obtained in four technical replicates of the experiment, presented in the form of plots of the relative distributions of the AFM-visualized objects with height.

All the curves shown in Figure 3 have their maxima at one and the same height *h_max1_* = (1.0 ± 0.2) nm. The curves corresponding to two of the four technical replicates (#2 and #4) also indicate an increased contribution of objects with 1.4 ± 0.2 nm height to the right wings of the respective *ρ(h)* distributions. According to the data obtained, the visualized objects were subsequently divided into two groups: objects with heights of up to 1.4 nm were attributed to “group 1”, while higher (*h* > 1.4 nm) objects were attributed to “group 2”. Hereinafter, the relative contents of the objects, attributed to either “group 1” or “group 2”, are designated as *α*_1_ and *α_2_,* respectively. The *α* values are expressed in %. Table 1 summarizes the results of processing AFM images obtained in this step of our study. The values of maximum heights *h_max1_* and *h_max2_,* and relative contents of objects *α_1_* and *α_2_* were determined after approximation of the *ρ(h)* distributions shown in Figure 3 using Equation (2).

**Figure 4:**
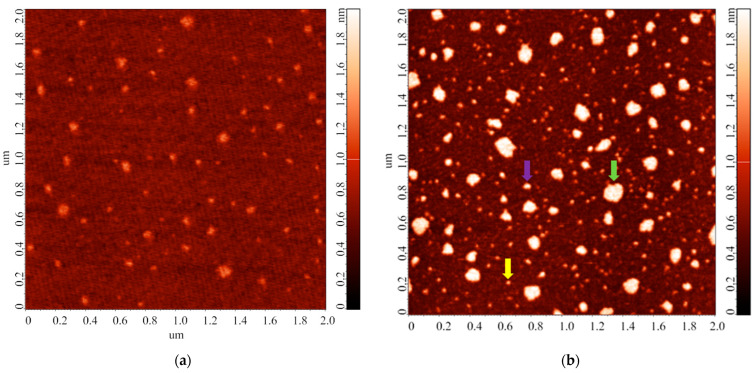

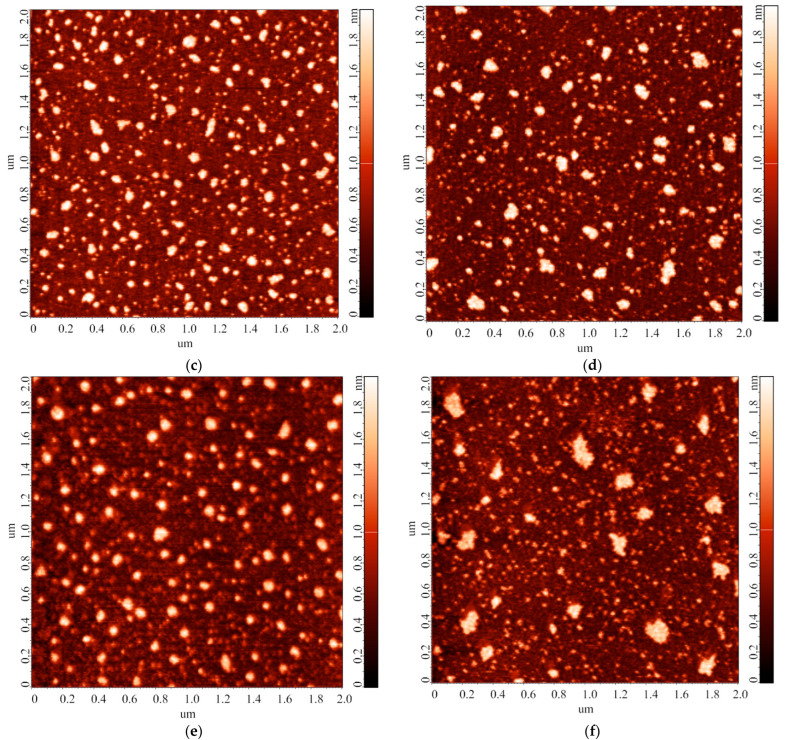
Typical AFM images of objects adsorbed onto mica substrates upon their incubation in 0.1 µM HRP solution at various temperatures: 15 °C (**a**), 20 °C (**b**), 22 °C (**c**), 30 °C (**d**), 35 °C (**e**), and 40 °C (**f**). In (**b**), the yellow arrow indicates an example of an object attributed to “group 1” (compact, *h* ≤ 1.4 nm); the green arrow indicates an example of an object attributed to “group 2” (laterally extended, *h* > 1.4 nm); the violet arrow indicates an example of an object attributed to “group 2” (laterally compact, *h* > 1.4 nm). For all the AFM images shown, scan size is 2 µm × 2 µm, and Z scale is 2 nm.

In the second step of the study, a series of experiments with variations in the incubation temperature of the HRP solution were performed. The samples were prepared according to the procedure described in the Materials and Methods (series 1). Typical AFM images of the mica surface, obtained in this series of experiments, are shown in Figure 4.

**Figure 5:**
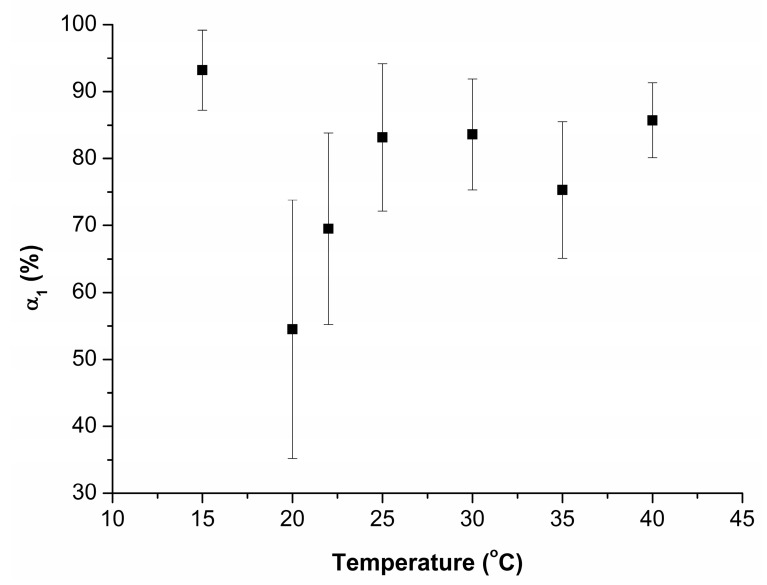
Temperature dependence of the relative content of AFM-visualized mica-adsorbed objects attributed to “group 1” (*α*_1_; *h* ≤ 1.4 nm).

The images shown in Figure 4 indicate that upon the incubation of the substrate in the 0.1 µM HRP solution at 15 °C (Figure 4a), the enzyme adsorbs onto mica in the form of compact objects with heights of ~1.2 nm. At higher temperature, the adsorbed objects are higher (with *h* up to 2 nm) and more extended laterally. Similar to the first step of the study, after processing the AFM data obtained, the contents of objects, attributed to either “group 1” (*α_1_*) or “group 2” (*α_2_*), were determined. Figure 5 displays the dependence of the relative content of objects, attributed to “group 1” (*α_1_*; *h* ≤ 1.4 nm), on the incubation temperature of the HRP solution.

The data presented in Figure 5 indicate that in all cases, mean values of *α*_1_ are equal to each other within the error. The values of *α_1_* (20 °C) and *α_1_* (22 °C) are, however, somewhat lower than those obtained at other temperatures. For these temperature points, the number of technical replicates is increased from three to six. The error for the respective mean values of *α_1_*, however, remains higher than those obtained under other experimental conditions.

### 3.3. Effect of Storage Duration of HRP Solution on Enzyme Aggregation State on Mica

**Figure 6:**
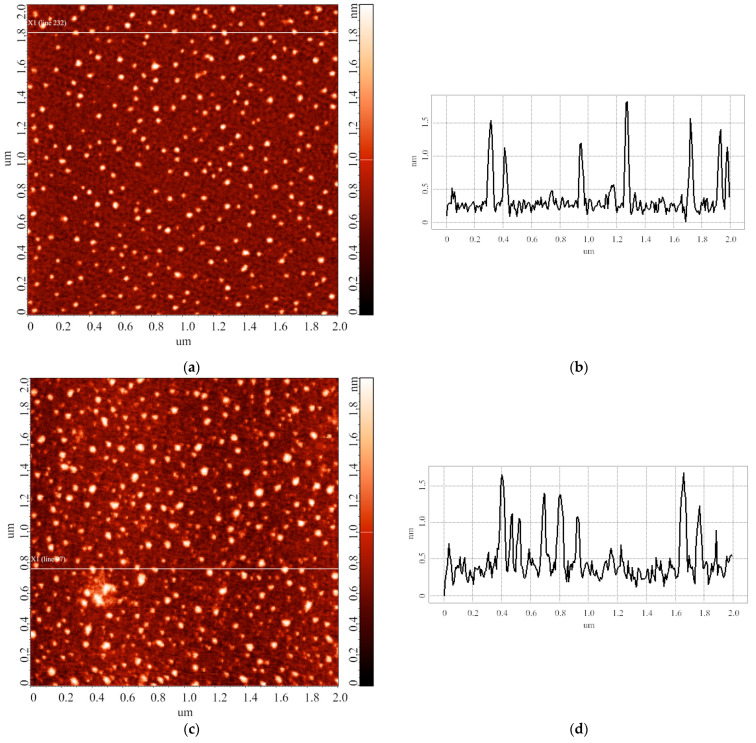

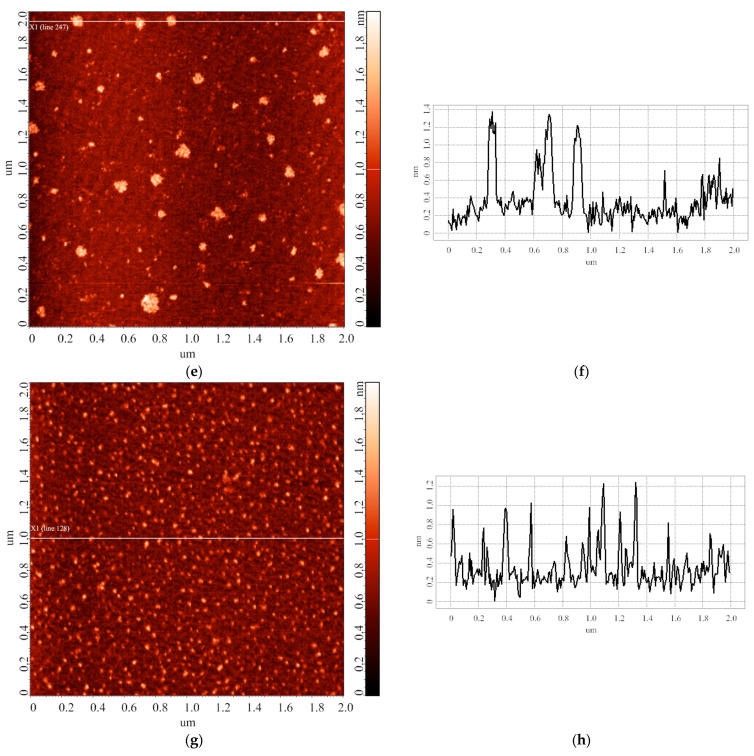
Typical AFM images (**left**) and cross-sectional profiles (**right**) of the surface of the mica substrates obtained after their incubation in HRP solutions stored for various time periods. Prior to the incubation, the solutions were stored for either 1 day (**a**,**b**), 7 days (**c**,**d**), 14 days (**e**,**f**), or 28 days (**g**,**h**). For all the AFM images shown, scan size is 2 µm × 2 µm, and Z scale is 2 nm.

In the next step of our study, we performed a series of experiments upon varying the duration of storage of the HRP solution before the incubation of mica substrates in it. The samples for this experimental series were prepared as described in the Materials and Methods (series 2). Figure 6 displays typical AFM images of the surface of the mica substrates obtained after their incubation in HRP solutions stored for various time periods prior to the incubation.

The images shown in Figure 6 indicate that the heights of the AFM-imaged objects make up to 2 nm. At that, both laterally compact (Figure 6c,g) and laterally extended (Figure 6c,e) are present on the substrate surface. It should be noted that a different adsorption efficiency is observed: namely, the number of objects visualized in the case of the solution stored for 28 days significantly exceeds that for the 14-day storage duration. Table 2 summarizes the results of processing the AFM data obtained in this experimental series: the relative contents of objects attributed to either “group 1” (*α_1_*; *h* ≤ 1.4 nm) or “group 2” (*α_2_*; *h* > 1.4 nm) are listed.

The data listed in Table 2 indicate that, with the increase in storage duration of the HRP solution, the relative content of objects, attributed to either “group 1” or “group 2”, remains constant within experimental error.

**Figure 7:**
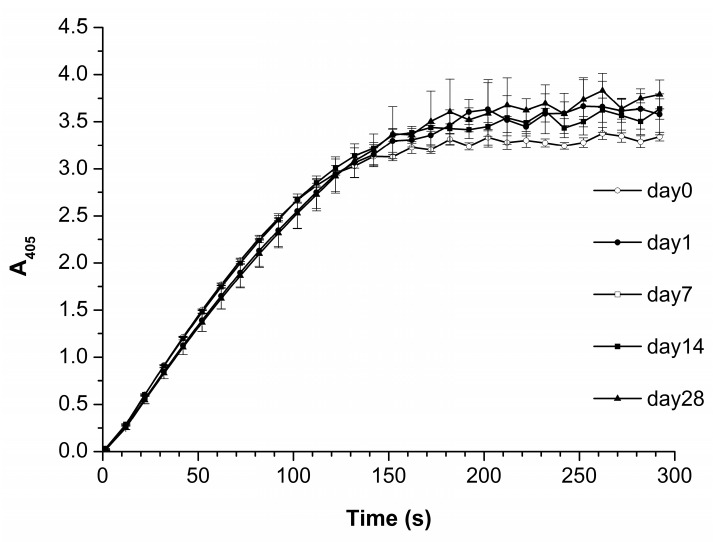
*A_405_(t)* curves obtained for HRP solution 1 h (white circles), 24 h (black circles), one week (white squares), two weeks (black squares), and one month (black triangles) after its preparation. The initial concentration of HRP solution was 10^−7^ M. The concentrations of HRP, ABTS, and H_2_O_2_ in the cell were 10^−9^ M, 0.3 mM, and 2.5 mM, respectively. Experimental conditions: cell pathlength 1 cm, temperature 25 °C, pH 5.0.

Figure 7 displays *A_405_(t)* curves obtained for HRP solutions stored for various time periods.

The *A_405_(t)* curves obtained indicate no influence of the storage duration of the HRP solution on the functional activity of the enzyme against the ABTS substrate.

## 4. Discussion

The molecular weight (*M_r_*) of HRP makes up 40 to 44 kDa [44,45]. Characteristic maximum heights *h_max_* for proteins with similar *M_r_* are as follows: putidaredoxin reductase, *h_max_* = 1.8 nm [23], *M_r_* = 45.6 kDa [46]; adrenodoxin reductase, *h_max_* = 1.8 nm [25], *M_r_* = 54 kDa [47]. In the blank experiments (Figure 1), we have obtained that the height of objects, nonspecifically adsorbed onto mica from protein-free solutions, does not exceed 0.8 nm. Accordingly, the objects with 1.0 nm and greater heights, visualized on the substrate surface, can be attributed to mica-adsorbed HRP [26]. These data coincide with previously reported values of the HRP height measured by AFM [26,48].

Upon the analysis of data obtained in the experimental series on the estimation of the influence of temperature of the incubation solution on the aggregation state of mica-adsorbed HRP, the HRP particles visualized by AFM on the mica substrate surface were divided into two groups. The results of experiments, performed at a HRP solution temperature of 25 °C, indicated that the height of objects attributed to “group 1” corresponded to 1.0 ± 0.2 nm (the relative content of these objects was α_1_ = 85.8 ± 9.6%). These objects can be attributed to the monomeric form of HRP adsorbed on mica. Objects with heights exceeding 1.4 nm were attributed to “group 2” (their relative content was *α_2_* = 14.2 ± 9.6%); these objects can be attributed to either HRP oligomers (dimers, trimers, etc.) or monomeric HRP, whose orientation on the substrate surface is different from that of monomers attributed to “group 1”. Upon adsorption onto a substrate surface, protein molecules are known to be able to become various orientations on the surface. Molecules with different orientation on the surface will accordingly have different heights measured by AFM [49]. The ratio between the relative contents of objects attributed to “group 1” and “group 2” allows one to make a conclusion regarding the change in the properties of a protein in its solution upon changing any external factor, as changes in the properties of biomolecules in solution affect both the oligomeric state of the protein in solution and the adsorption properties of individual biomolecules. In our study, two factors—temperature of the protein solution upon the incubation of the substrate in it, and the duration of storage of the protein solution prior to the incubation of the substrate—have been investigated, though other researchers can, however, use the proposed approach with regard to their scientific tasks.

In our experiments, the results obtained upon varying the incubation temperature indicated that with increasing the incubation temperature, the mean values of α_1_ coincide within the error. At 20 °C and 22 °C temperatures, the respective mean values of α_1_, however, tend to be somewhat lower than those at other temperatures (Figure 5). Accordingly, the relative content of monomers in the solution decreases, while the proportion of objects attributed to “group 2” increases. At that, an increased number of technical replicates performed at these temperatures did not help us to decrease the α_1_ error, which remained greater than that obtained at other temperatures. In our opinion, this is an interesting fact indicating an “instability” of the biomolecular system at 20 °C and 22 °C. The latter can ultimately affect its functional properties. Similar results—namely, changes in the aggregation state and functional properties of a protein upon only a few degrees shift in temperature—were reported previously for other enzymes. Thus, in [50], AFM experiments with cytochrome P450 BM3 indicated a decrease in its aggregation state (and, accordingly, an increase in the content of monomers) upon increasing the incubation temperature from 10 to 22 °C. With the further increasing in temperature of the BM3 solution to 37 °C, the data obtained by fluorescent analysis indicated further changes in the microenvironment of the aromatic residues in this protein. At 33 °C, AFM revealed that the change in the microenvironment of aromatic residues from hydrophobic to hydrophilic causes a slight increase in the degree of protein aggregation. This leads to the loss of functional activity [51,52,53].

Our experiments on the estimation of the influence of storage duration of the protein solution on the properties of the protein biomolecules in solution indicated that with the increase in the storage duration, the relative content of objects, attributed to either “group 1” or “group 2”, remains unchanged within the error (Table 2). Accordingly, one can state that, in terms of the adsorption behavior and aggregation state of biomolecules, the HRP solution is relatively stable for at least 28 days. Here, we use the term “relative stability”, as, according to the AFM images obtained (Figure 6e–g), in the case of storage of the solution for 14 and 28 days, a change in the lateral dimensions of the visualized objects and in the adsorption efficiency (that is, the number of visualized objects per unit area) was observed. Currently, the lateral dimensions of objects on the substrate surface cannot be measured as precisely as their height, due to the artifact-inducing effect caused by the curvature of the AFM probe [54]. In perspective, this problem can be solved by using specialized software for AFM data processing. However, at present, changing the lateral properties can only be used as a “qualitative” parameter, and in our study, this parameter indicated that during long storage periods, changes in the structure of biomolecules in the protein solution take place. These changes are manifested in the tendency of biomolecules to assemble into agglomerates upon their adsorption onto the substrate surface: the heights of the agglomerates correspond to that of the monomeric form of the protein, but increased lateral dimensions are observed. At that, their functional activity remains unaffected. Spectrophotometry data (Figure 7) indicate no change in the HRP enzymatic activity for all storage durations studied, indicating that the structural changes registered by AFM do not involve the active site of the biomolecule. Other researchers [55] demonstrated that highly concentrated solutions of urease in phosphate buffer can be stored at 4 °C for 28 days without any significant loss of activity, which is of practical importance. In [56], the effect of storage conditions on antibody affinity was analyzed by ELISA. No significant change in the affinity of the antibody-HRP conjugates against their respective antigens was observed after 5 months of storage in StabilGuard1 buffer at 4 °C. Thus, our experiments also indicated that the enzymatic activity of HRP can be retained for a long time despite structural changes at the level of its single biomolecules.

## 5. Conclusions

The AFM-based technique for the estimation of properties of HRP macromolecules in solution has been proposed. The characteristics of the macromolecules adsorbed from their solution onto the mica surface indicate changes in their properties occurring in the solution upon varying either the temperature or the storage duration of the solution. With regard to studying biological systems, the AFM-based approach proposed herein has a number of advantages, as the sample preparation is simple, and the experimental conditions are similar to those used in other methods.

AFM tools and devices are fabricated by a wide range of manufacturers. Thus, in our opinion, the proposed approach will be useful for other scientific groups.

## Figures and Tables

**Table 1:** Results of processing the data on AFM imaging of objects adsorbed on mica substrates upon their incubation in 0.1 µM HRP solution at room temperature (*T* = 25 °C).

Technical Replicate	Group 1		Group 2	
	*h_max1_*, nm	α_1_, %	*h_max2_*, nm	α_2_, %
1	1 ± 0.2	93.7	-	6.3
2		79.8	1.4 ± 0.2	20.2
3		96.4	-	3.6
4		73.2	1.5 ± 0.2	26.8
Mean value	1 ± 0.2	85.8 ± 9.6	1.45 ± 0.2	14.2 ± 9.6

**Table 2:** Results of processing the AFM visualization data of objects adsorbed on the mica surface after incubation in an HRP solution stored for various time periods.

Storage Duration	“Group 1”, *α_1_* %	“Group 2”, *α_2_* %
1 day	88.5 ± 9.3	11.5 ± 9.3
7 days	92.6 ± 5.5	7.4 ± 5.5
14 days	88.3 ± 9.1	11.7 ± 9.1
28 days	89.3 ± 6.7	10.7 ± 6.7

## Data Availability

Data is contained within the article. The data underlying this manuscript can be obtained from the corresponding author (T.O.P.) upon reasonable request.
